# *Penaeus monodon* IKKs Participate in Regulation of Cytokine-Like System and Antiviral Responses of Innate Immune System

**DOI:** 10.3389/fimmu.2019.01430

**Published:** 2019-06-25

**Authors:** Zittipong Nhnhkorn, Piti Amparyup, Taro Kawai, Anchalee Tassanakajon

**Affiliations:** ^1^Faculty of Science, Department of Biochemistry, Center of Excellence for Molecular Biology and Genomics of Shrimp, Chulalongkorn University, Bangkok, Thailand; ^2^National Center for Genetic Engineering and Biotechnology (BIOTEC), National Science and Technology Development Agency (NSTDA), Pathumthani, Thailand; ^3^Laboratory of Molecular Immunobiology, Division of Biological Science, Graduate School of Science and Technology, Nara Institute of Science and Technology, Nara, Japan

**Keywords:** antiviral responses, IKK-NF-κB signaling cascade, *Penaeus monodon*, white spot syndrome virus, shrimp immunity

## Abstract

The IKK-NF-κB signaling cascade is one of the crucial responsive mechanisms in inflammatory and immune responses. The key kinase proteins called inhibitor of kappa B kinases (IKKs) serve as the core elements involved in cascade activation. Here, the complete ORFs of IKK homologs, *PmIKKβ*, *PmIKKε1*, and *PmIKKε2*, from the black tiger shrimp *Penaeus monodon* were identified and characterized for their functions in shrimp antiviral responses. The *PmIKK* transcripts were widely expressed in various examined tissues and the *Pm*IKKε protein was detected in all three types of shrimp hemocytes. Only the *PmIKKε1* and *PmIKKε2* were responsive to white spot syndrome virus (WSSV), yellow head virus (YHV) and a bacterium *Vibrio harveyi* infection, while the *PmIKKβ* exhibited no significant response to pathogen infection. On the contrary, suppression of *PmIKKβ* and *PmIKKε* by dsRNA-mediated RNA interference (RNAi) resulted in a rapid death of WSSV-infected shrimp and the significant reduction of an IFN-like *PmVago4* transcript. Whereas, the mRNA levels of the antimicrobial peptides, *ALFPm3* and *CrustinPm5*, and a transcription factor, *PmDorsal* were significantly increased, those of *ALFPm6, CrustinPm1, CrustinPm7, PmVago1, PmRelish*, and *PmCactus* were unaffected. Overexpression of *Pm*IKKβ and *Pm*IKKε in HEK293T cells differentially activated the NF-κB and IFNβ promoter activities, respectively. These results suggest that the *Pm*IKKβ and *Pm*IKKε may act as common factors regulating the expression of immune-related genes from various signaling pathways. Interestingly, the *Pm*IKKs may also contribute a possible role in shrimp cytokine-like system and cross-talking between signaling transductions in innate immune responses.

## Introduction

Innate immune system represents the first line of defense responding against the invading pathogens. The various defense mechanisms therein are stimulated when the pathogen-associated molecular patterns (PAMPs) are recognized by host receptors such as Toll-like receptors (TLRs), retinoic acid-inducible gene-I (RIG-I)-like receptors (RLRs), NOD-like receptors (NLRs), C-type lectin receptors (CLRs), or cytosolic DNA sensors (1, 2). The recognition of PAMPs induces the activation of cellular and humoral immune responses through the recruitment of immune molecules aiming to destroy the invading pathogens (3–5). In vertebrates, the PAMPs are recognized by pattern recognition receptors (PRRs) and trigger the IKK-NF-κB pathway which includes inhibitor of kappa B kinase (IKK) and transcription factor designated nuclear factor kappa B (NF-κB). The pathway has an essential role in coordinating the expression of type I interferons (IFNs), pro-inflammatory cytokines and chemokines. Following the activation, the elimination of invading viruses and bacteria commences by initiating innate and adaptive immune systems (6–8).

The recruitment of transcription factor NF-κB is achieved when its cytoplasmic inhibitor protein namely, inhibitor of kappa B alpha (IκBα) is phosphorylated by signal-induced phosphorylation and involved in the degradative polyubiquitination (9, 10). Two inhibitors of kappa B kinases (IKKα and IKKβ) are required for mediating the phosphorylation of IκBα and function to connect among the cascades of most signal transduction pathways (11, 12). The degradation of the phosphorylated IκBα subsequently releases the NF-κB protein which in turn translocates into the nucleus, thereby, activating the expression of a series of genes required for immune responses (12, 13). In addition, NF-κB, interferon β (IFNβ) and IFN regulatory factor 3/7 (IRF3/7) are stimulated simultaneously following the recognition of extrinsic dsRNA by Toll-like receptors 3 (TLR3), TLR4 and RIG-I (14, 15). Several essential peptides or interferons in immune system are characterized and regulated via NF-κB transcription factor in responses to different stimuli (7).

Toll and immune deficiency (IMD) pathways are the two widely studied signaling pathways in fruit fly *Drosophila* which recognize the pathogen signature molecules and respond through the production of antimicrobial peptides (AMPs) (16). In the IMD pathway, several IKK family proteins are recently reported to facilitate the clearance of microbial pathogens via interaction of IκBα and a transcription factor named p65 (IκBα-p65-) and p100-p52-independent mechanism (17, 18). The p100-like NF-κB precursor protein or Relish is phosphorylated by IKKβ and acts as a central component in the IMD pathway to promote the expression of immune-related genes (7, 17). During the infection of Gram-negative and certain Gram-positive bacteria, the activated p100-like transcription factor in NF-κB family proteins called Relish further stimulates the expression of antimicrobial peptides (AMPs) (11). In *Drosophila*, stimulation by fungi and Gram-positive bacteria also activates DIF and Dorsal, two p65-like NF-κB proteins through the phosphorylation of an IκB-related inhibitor Cactus in Toll pathway (16). However, the mechanism by which the effector kinase facilitate the phosphorylation cascade is still unrevealed.

In the IMD pathway, the Relish cascade relies on an active IKK complex, which comprises IKKβ and a complex of IKKγ and NF-κB essential modulator (IKKγ/NEMO). Once activated by TAK1, the IKK complex phosphorylates the NF-κB protein Relish on specific serine residues releasing its regulatory domain to direct the expression of dedicated genes (7, 16, 17). Moreover, IKKβ from *Drosophila melanogaster* (*Dm*IKKβ) participates in immune responses through the IMD pathway but not the Toll pathway. The *Dm*IKKε is not involved in NF-κB activation directly but regulates non-apoptotic caspases through the degradation of *Drosophila* inhibitor of apoptosis protein 1 (DIAP1) (10, 19). In addition, *Shigella* bacteria modify the host ubiquitin ligase to prohibit tumor-necrosis factor (TNF) receptor-associated factor 6 (TRAF6) auto-polyubiquitination in NF-κB pro-inflammatory pathway to prolong the infection. This modification suggests the importance of NF-κB signaling pathway against microbial infection (20). To date, whether these detailed mechanisms exist in crustaceans and how they contribute in shrimp innate immune system are still of great interest. Recent report in Pacific white shrimp, *Litopenaeus vannamei* revealed the activation of Vago gene (*Lv*Vago) by IFN regulatory factor (*Lv*IRF) in promoter luciferase assay (21). The expression of Vago was increased after viral infection and secreted as an interferon in arthropods (22). Therefore, it is possible that the Vago might act as a IFN-like molecule in shrimp antiviral response similar to the interferon from vertebrates.

Thus far, the mechanisms of shrimp innate immune system for pathogen responses including the correlation of IκB kinases between Toll and IMD pathways are still unclear. Here, we identified three IκB kinases from *P*. *monodon* (*Pm*IKKβ, *Pm*IKKε1, and *Pm*IKKε2) and characterized their involvement in pathogen responses. Groups of immune genes related to the expression of *PmIKK*s were indicated using dsRNA-mediated *in vivo* gene silencing. Subsequently, their possible roles in shrimp IFN-like system were determined by luciferase assay. The results revealed the potential roles of *Pm*IKKs in shrimp antiviral response with an IFN-like system through *Pm*Vago.

## Materials and Methods

### Experimental Shrimp

Healthy black tiger shrimp, *P*. *monodon*, were purchased from a local shrimp farm with the average body mass of 10–15 g for gene expression analysis and that of 3–5 g for dsRNA-mediated RNA interference experiments. Shrimp were cultivated in recirculating aquaria filled with air-pumped seawater with a salinity of 20%0 at an ambient temperature of about 29 ± 1°C. They were fed with a commercial diet twice a day for at least 7 days for acclimation before experiments. This study was conducted under the ethical principles and guidelines according to the animal use protocol approved by Chulalongkorn University Animal Care And Use Committee (CU-ACUC).

### RNA Extraction and cDNA Synthesis

Tissue samples from healthy or pathogen-infected shrimp were homogenized in 1 ml GENEzol™ Reagent (Geneaid Biotech). The total RNA was isolated and treated with DNase I (RNase-free) (NEB). One microgram RNA was reverse transcribed in single-stranded cDNA synthesis using RevertAid First Strand cDNA Synthesis Kit (Thermo Scientific) as described in the manufacturer's instructions. The RNA extract and cDNA were stored in −80°C until use.

### Cloning and Nucleotide Sequencing of *Pm*IKKβ and *Pm*IKKε

The open reading frame (ORF) of *PmIKKβ* was obtained from the hemocyte cDNA by PCR amplification using specific primers (Table 1) based on a partial sequence of *PmIKKβ* (accession no. PM53485) from expressed sequence tag (EST) database of *Penaeus monodon* (http://pmonodon.biotec.or.th) (23) and *LvIKK*β (accession no. JN180642) from *Litopenaeus vannamei*. The hemolymph was drawn from shrimp ventral sinus with 10% (w/v) tri-sodium citrate solution and centrifuged at 800 × *g*, 4°C for 10 min to collect the hemocytes for total RNA extraction and synthesis of the cDNA template.

**Table 1:** List of nucleotide primers used in the experiments.

**Primer name**	**Sequence (5^′^ → 3^′^)**	**Purpose**
ORF-PmIKKβ-F	GGTGTGAGGTGCAACATGGCA	ORF cloning
ORF-PmIKKβ-R	TCAGCAGAAGACTACAAGGAAGTT	ORF cloning
ORF-PmIKKε-F	ACCGTCTCGAGAAAAGGGTCCTA	ORF cloning
ORF PmIKKε-R	TCCGTCTGGACTCGCTGGACT	ORF cloning
IKKεRACE3′-F	GAAACCCTCCTGGCCTCCGTCACAG	RACE
IKKεRACE3′Ne-F	CAGGACACCTTAGGCAATACAGAG	RACE
EF-1α-F	GGTGCTGGACAAGCTGAAGGC	qRT-PCR and RT-PCR
EF-1α-R	CGTTCCGGTGATCATGTTCTTGA	qRT-PCR and RT-PCR
RT-PmIKKβ-F	CTGAGGGCATGACGCGACCAC	qRT-PCR and RT-PCR
RT-PmIKKβ-R	GCCTGCTCATCATAGTAGTCGAG	qRT-PCR and RT-PCR
RT-PmIKKε-F	ACCGTCTCGAGAAAAGGGTCCTA	qRT-PCR and RT-PCR
RT-PmIKKε-R	CGGATCGTCCAGAATGTTGAAGAG	qRT-PCR and RT-PCR
PmRelish-F	TCTCCAGGTGAGCACTCAGTTGGC	qRT-PCR
PmRelish-R	GCTGTAGCTGTTGCTGTTGTTGAG	qRT-PCR
ALFPm3-F	CCCACAGTGCCAGGCTCAA	qRT-PCR
ALFPm3-R	TGCTGGCTTCTCCTCTGATG	qRT-PCR
ALFPm6-F	AGTCAGCGTTTAGAGAGGTT	qRT-PCR
ALFPm6-R	GCTCGAACTCTCCACTCTC	qRT-PCR
CrustinPm1-F	CTGCTGCGAGTCAAGGTATG	qRT-PCR
CrustinPm1-R	AGGTACTGGCTGCTCTACTG	qRT-PCR
CrustinPm5-F	ACCAGGGCCAAGGAAACTAT	qRT-PCR
CrustinPm5-R	GCAGCATTTGTCGTTTGAGG	qRT-PCR
CrustinPm7-F	GGCATGGTGGCGTTGTTCCT	qRT-PCR
CrustinPm7-R	TGTCGGAGCCGAAGCAGTCA	qRT-PCR
PmVago1-F	GAACACACCCCAGTGCACTGGT	qRT-PCR
PmVago1-R	ATGGAGCTTGTTCCCCTTCTGTG	qRT-PCR
PmVago4-F	ACTCCTCTCCCTTCAGGGCATC	qRT-PCR
PmVago4-R	TGGCAGGAACTTCTCTCGCTGC	qRT-PCR
PmDorsal-F	TCACTGTTGACCCACCTTAC	qRT-PCR
PmDorsal-R	GGAAAGGGTCCACTCTAATC	qRT-PCR
PmCactus-F	ACGGCAGTAGGATCGGGGTTTGCCT	qRT-PCR
PmCactus-R	ATGCCCCACAGAGGTGATGCCCTGA	qRT-PCR
VP28-F	GGGAACATTCAAGGTGTGGA	qRT-PCR
VP28-R	GGTGAAGGAGGAGGTGTTGG	qRT-PCR
dsPmIKKβ-F	GAATGGATGAAGCGTGTACGCAC	RNAi
dsPmIKKβ-R	ACTGTCACGTGCAACCCACTGCT	RNAi
dsPmIKKβ-T7-F	TAATACGACTCACTATAGGGAATGGATGAAGCGTGTACGCAC	RNAi
dsPmIKKβ-T7-R	TAATACGACTCACTATAGGACTGTCACGTGCAACCCACTGCT	RNAi
dsPmIKKε-F	AATAGGTGTGACACTTTACCACGT	RNAi
dsPmIKKε-R	TGGTTGACTGGATTCATGTCTGTC	RNAi
dsPmIKKε-T7-F	TAATACGACTCACTATAGGAATAGGTGTGACACTTTACCACGT	RNAi
dsPmIKKε-T7-R	TAATACGACTCACTATAGGTGGTTGACTGGATTCATGTCTGTC	RNAi
dsGFP-F	AGTGCTTCAGCCGCTACCC	RNAi
dsGFP-R	GCGCTTCTCGTTGGGGTC	RNAi
dsGFP-T7-F	TAATACGACTCACTATAGGAGTGCTTCAGCCGCTACCC	RNAi
dsGFP-T7-R	TAATACGACTCACTATAGGGCGCTTCTCGTTGGGGTC	RNAi

The ORFs of *PmIKKε1* and *PmIKKε2* were obtained by a RACE-PCR approach using SMARTer™ RACE cDNA Amplification Kit (Takara Bio) and primers designed from a partial sequence of EST *PmIKKε* (accession no. PM42457). The PCR reaction was performed using 2.5 μl cDNA template in 50 μl reaction volume containing 1X Advantage 2 PCR Buffer, 1X dNTP Mix (0.2 mM each), 1X Universal Primer A Mix (UMP), 0.2 μM gene-specific primer (GSP) and 1X Advantage 2 Polymerase Mix (Takara Bio). The reaction was carried out with following cycling conditions; 5 cycles of 94°C for 30 s and 72°C for 3 min followed by 5 cycles of 94°C for 30 s, 70°C for 30 s, and 72°C for 3 min and 25 cycles of 94°C for 30 s, 68°C for 30 s, and 72°C for 3 min. The nucleotide sequences of *PmIKK*β, *PmIKK*ε*1* and *PmIKKε2* were cloned using RBC T&A Cloning Kit (RBC) and sequenced by Macrogen, Korea with M13 universal and M13 reverse primers.

### Bioinformatics Analysis

Nucleotide and protein sequence similarities of IKK and IKK family genes were analyzed with GENETYX 7.0.3 (GENETYX Corporation) and BLAST® algorithm at the National Center for Biotechnology Information (https://blast.ncbi.nlm.nih.gov/Blast.cgi). Multiple sequence alignments were performed using Clustal Omega (https://www.ebi.ac.uk/Tools/msa/clustalo/). The amino acid sequences of *Pm*IKKβ, *Pm*IKKε1, and *Pm*IKKε2 were deduced by ExPASy-Translate tool (https://web.expasy.org/translate/) and analyzed for protein motif features using Simple Modular Architecture Research Tool, SMART 8.0 (http://smart.embl-heidelberg.de/). The neighbor joining phylogenic tree was constructed in MEGA 7.0 software (http://www.megasoftware.net/index.html) based on the amino acid sequences of IKK and IKK-family proteins in invertebrate and vertebrate species. Bootstrap sampling was reiterated for 1,000 times.

### Tissue Distribution Analysis of *PmIKKβ, PmIKKε1*, and *PmIKKε2*

The expression of *PmIKKβ*, *PmIKKε1* and *PmIKKε2* in different shrimp tissues including hemocyte, lymphoid organ, gill, hepatopancreas, heart, intestine, muscle, eyestalk and stomach was examined. The selected tissues were collected individually from three healthy shrimp for total RNA extraction and pooled for cDNA synthesis. Semi-quantitative RT-PCR reaction was performed using specific primers (Table 1) in 25 μl reaction volume containing 1 μl of first-stand cDNA, 1X Reaction Buffer (50 mM KCl, 1.5 mM MgCl_2_, 10 mM Tris-HCl (pH 8.3), 0.1 mg/ml BSA, 10 mM (NH_4_)_2_SO_4_, 0.1 μM dNTP mix, 0.2 μM forward and reverse primers and 1.25 units of RBC *Tag* DNA polymerase (RBC). The reaction was carried out under the following conditions; 1 cycle of 94°C for 2 min followed by 30 cycles of 94°C for 30 sec, 55°C for 30 s and 72°C for 30 s and the final extension at 72°C for 7 min. The elongation factor-1α gene (*EF-1α*) was used as an internal control. The amplified PCR products were analyzed by 2% (w/v) agarose-TBE gel electrophoresis and visualized by UV-transillumination.

### Immunofluorescence Microscopy of *Pm*IKKβ and *Pm*IKKε in Shrimp Hemocytes

Protein expression of *Pm*IKKβ and *Pm*IKKε in shrimp hemocytes was detected using the monoclonal mouse antibody specific to human IKKε (Abcam) and monoclonal rabbit antibody specific to human IKKβ (Invitrogen). Total hemolymph was drawn from healthy *P. monodon* (8–10 g) and fixed with 4% paraformaldehyde at a 1:1 ratio. The hemocytes were separated by centrifugation and resuspended in 1X phosphate buffered saline, pH 7.4 (1X PBS) before counting with a hemocytometer. Cells were mounted on the poly-L-lysine coated-coverslips in a 24-well plate and washed three times with 0.02% Triton® X-100 in 1X PBS followed by permeabilization with 1X PBS containing 100 mM glycine and 0.02% Triton® X-100 for 30 min. Cells were then washed three times and blocked with 1X PBS containing 10% FBS and 0.02% Triton® X-100 at 4°C, overnight. Cells were washed again and probed with anti-IKKβ or anti-IKKε primary antibody in 1X PBS containing 10% FBS and 0.02% Triton® X-100 at 4°C overnight, followed by washing and incubation with anti-mouse or anti-rabbit secondary antibody conjugated with Alexa Fluor® 488 at room temperature for 2 h. The nuclei were stained with Hoechst 33342 (Thermo Scientific). The coverslips were mounted with Fluoro-KEEPER Antifade Reagent (Nacalai Tesque) and sealed on glass slides. Fluorescence images were detected by LSM 700 laser scanning confocal microscope (Carl Zeiss).

### Preparation of *PmIKKβ* and *PmIKKε* Double-Stranded RNAs and *in vivo* Silencing Efficiency by dsRNA-Mediated RNA Interference (RNAi)

The double-stranded RNAs (dsRNAs) corresponding to *PmIKKβ*, *PmIKKε*, and *GFP* sequences (ds*PmIKKβ*, ds*PmIKKε*, and ds*GFP*, respectively) were synthesized by *in vitro* transcription using T7 RiboMAX™ Express Large Scale RNA Production System (Promega) according to the manufacturer's protocol. The DNA templates for *in vitro* transcription of sense and antisense RNA strands were amplified separately by PCR reactions containing gene specific primers attached with T7 RNA polymerase binding site (Table 1). The ds*GFP* for a negative control was amplified from pEGFP-1 vector (Clonetech) harboring green fluorescent protein (*GFP*) gene. The complementary single-stranded RNAs were annealed to generate the double-stranded RNAs. The dsRNAs were quantified and stored at −80°C until use.

To verify the silencing efficiency of dsRNAs *in vivo*, shrimp were doubly injected with ds*PmIKKβ* or ds*PmIKKε* (10 μg/g shrimp) dissolved in 150 mM NaCl by intramuscular injection, whereas the control group was doubly injected with ds*GFP*. At 24 h after the second dsRNA injection, the hemolymph was drawn for total hemocyte RNA extraction and first strand cDNA synthesis. Transcript levels of *PmIKKβ* and *PmIKKε* were analyzed by quantitative RT-PCR (qRT-PCR). The expression of elongation factor-1α gene (*EF-1α*) was used as an internal control.

### Expression Profiles of *PmIKKβ, PmIKKε1*, and *PmIKKε2* in Response to Viral and Bacterial Challenges and *PmIKK* Silencing Effects on Immune-Related Genes After WSSV Infection

In microbial challenges, healthy shrimp were injected intramuscularly in the third abdominal segment with 30 μl of phosphate-buffered saline (1X PBS; 137 mM NaCl, 2.7 mM KCl, 8 mM Na_2_HPO_4_, and 2 mM KH_2_PO_4_, pH 7.4) as a control, 1 × 10^5^ copies of purified WSSV, 1 × 10^5^ copies of purified YHV, and 1 × 10^6^ CFU/ml of *V. harveyi* 639 inoculum for immune challenge experiments. Three shrimp from each group were randomly collected at 0, 6, 12, 24, 48 h post injection (hpi). Shrimp total hemocyte was collected for RNA isolation, and cDNA preparation for qRT-PCR analysis.

To examine the silencing effects of *PmIKKβ* and *PmIKKε* on immune-related genes, dsRNA-mediated gene silencing was performed. The shrimp were doubly injected intramuscularly with 10 μg/g shrimp of ds*GFP* for control group, ds*PmIKKβ* and ds*PmIKKε* dissolved in 150 mM NaCl. Following gene silencing, shrimp were injected with 1X PBS and 1 × 10^5^ copies of purified WSSV at 24 h post the second dsRNA injection. Three shrimp per a treatment group were randomly collected at 24 h post infection for total RNA extraction and cDNA synthesis. The expression profiles of immune-related genes including *PmVago1, PmVago4, PmCactus, PmDorsal, PmRelish, ALFPm3, ALFPm6, CrustinPm1, CrustinPm5*, and *CrustinPm7* were determined by qRT-PCR using specific primers (Table 1).

The reactions were performed in 20 μl volume containing 1 μl of cDNA template, 10 μl of 2X Luna® Universal qPCR Master Mix (NEB) and 0.25 μM primer mix using CFX96 Touch™ Real-Time PCR Detection System (Bio-Rad). Quantitative RT-PCR was carried out with the following cycling parameters; 1 cycle of 95°C for 1 min followed by 40 cycles of 95°C for 15 s and 60°C for 30 sec. The expression of elongation factor-1α gene (*EF-1α*) was used as an internal control. Melt curve analysis was performed at the end of PCR thermal cycle for determining the specificity of amplification. The reactions were carried out in triplicates. The relative expression of *PmIKK*s was calculated using a comparative method described by Pfaffl (2001). The data were shown as means ± standard deviations (SD). Statistical analysis was performed using one-way ANOVA followed by Duncan's new multiple range test. The data was considered for statistical differences with the significance at *P* < 0.05.

### Survival Rates of *PmIKKβ*- and *PmIKKε*-Silenced Shrimp After WSSV Infection

To further investigate the roles of *Pm*IKKβ and *Pm*IKKε upon WSSV infection, groups of 10 shrimp (3–5 g) were doubly injected with 10 μg/g shrimp of *in vitro*-transcribed ds*PmIKKβ*, ds*PmIKKε*, ds*GFP*, and 30 μl of 150 mM NaCl with an interval of 24 h. At 6 h following the second dsRNA injection, shrimp were injected intramuscularly with 1 × 10^5^ copies of purified WSSV or 1X PBS, pH 7.4 for immune challenge. The cumulative mortalities were recorded daily for 10 days after WSSV infection. The experiment was carried out in triplicates. Statistical analysis was performed using one-way ANOVA with the significance at *P* < 0.05.

### Quantification of WSSV Copy Number in *PmIKKβ*- and *PmIKKε*-Silenced Shrimp

To study the effect of *PmIKKβ* and *PmIKKε* suppression on WSSV replication, the copy number of WSSV in *PmIKKβ*- and *PmIKKε*-silenced shrimp was determined. The shrimp were doubly injected with ds*GFP*, ds*PmIKK*β and ds*PmIKK*ε followed by 1 × 10^5^ copies of purified WSSV at 24 h after second dsRNA injection. Shrimp genomic DNA was extracted from gills at 120 hpi using FavorPrep™ Tissue Genomic DNA Extraction Mini Kit (Favorgen). Total genomic DNA was quantified by NanoDrop™ 2000c Spectrophotometer (Thermo Scientific) and used as DNA template for viral copy number analysis. Quantitative RT-PCR was performed in triplicates using Luna® Universal qPCR Master Mix (NEB) with 1 μl genomic DNA (15 ng/μl) and VP28 primers (Table 1). The cycling condition was performed with 95°C for 1 min, followed by 40 cycles of 95°C for 15 s and 60°C for 30 s. The recombinant plasmid containing a conserved region of WSSV *VP28* gene was used to generate a standard curve.

### Cell Culture and Luciferase Reporter Assay

To evaluate the synergy of *Pm*IKKβ, *Pm*IKKε1, and *Pm*IKKε2 with shrimp cytokine-like system and NF-κB signaling, luciferase reporter assay was performed. HEK293T cells were purchased from the CH3 BioSystems and cultured in Dulbecco's modified Eagle's medium (Nacalai Tesque) supplemented with 10% fetal bovine serum (Invitrogen) at 37°C in a humidified 5% CO_2_/95% air atmosphere. The cDNA fragments coding *PmIKKβ*, *PmIKKε1*, and *PmIKK*ε*2* were cloned into pcDNA3-Myc to generate pcDNA-*Pm*IKKβ-Myc, pcDNA-*Pm*IKKε1-Myc, and pcDNA-*Pm*IKKε2-Myc protein expression plasmids, respectively. The pGL3-IFNβ luciferase reporter plasmid for IFN-β was constructed by cloning a fragment of murine IFN-β promoter region (−125 to +55) as described previously. (24) The endothelial cell-leukocyte adhesion molecule (ELAM)-1 promoter-derived luciferase reporter plasmid (pGL3-NF-κB luciferase reporter) has been prepared (14). The insert cDNAs of all constructs were confirmed using BigDye® Terminator v3.1 (Thermo Scientific) in an ABI PRISM Genetic Analyzer (Applied Biosystems).

For reporter assays, human embryonic kidney 293T (HEK293T) cells (5 × 10^4^ cells/well) seeded on 24-well plates for 24 h were transiently co-transfected with 50 ng of pGL3-IFNβ or pGL3-NF-κB luciferase reporter plasmids and 1 μg each of protein expression plasmids or empty control plasmid using polyethylenimine (PEI) at a ratio of 1:3 (μg:μl) in Opti-MEM (Life Technologies). As an internal control, 10 ng of pRL-TK *Renilla* luciferase reporter plasmid was transfected simultaneously. Twenty-four hours after transfection, cells were harvested and lysed for the assessment of protein expression and luciferase reporter assay using Dual-Glo® Luciferase Assay System (Promega) according to the manufacturer's instructions. Luciferase activities were measured using a TriStar^2^ LB 942 Modular Multimode Microplate Reader (Berthold).

## Results

### Cloning and Sequence Characterization of *Pm*IKKβ and *Pm*IKKε

Two partial nucleotide sequences related to IκB kinases (IKKs) including *PmIKKβ* (753 bp) and *PmIKKε* (380 bp) were retrieved from the *P. monodon* EST database. The complete ORFs of *PmIKKβ* and *PmIKKε* were obtained by PCR amplification using specific primers (Table 1) and cDNA template prepared from healthy shrimp hemocytes. Analysis of nucleotide sequences in ExPaSy bioinformatics resource demonstrated that a 2,376 bp of *PmIKKβ* ORF (accession no. MK331816) encoded a 791-amino acid protein (Supplementary Figure 1A) with predicted molecular mass of 89.37 kDa and isoelectric point (pI) of 7.56. Sequence analysis using BLAST^®^ from the NCBI database showed 95% and 30% identity with Pacific white shrimp *Litopenaeus vanamei* IKKβ and *Drosophila* IKKβ, respectively.

**Figure 1:**
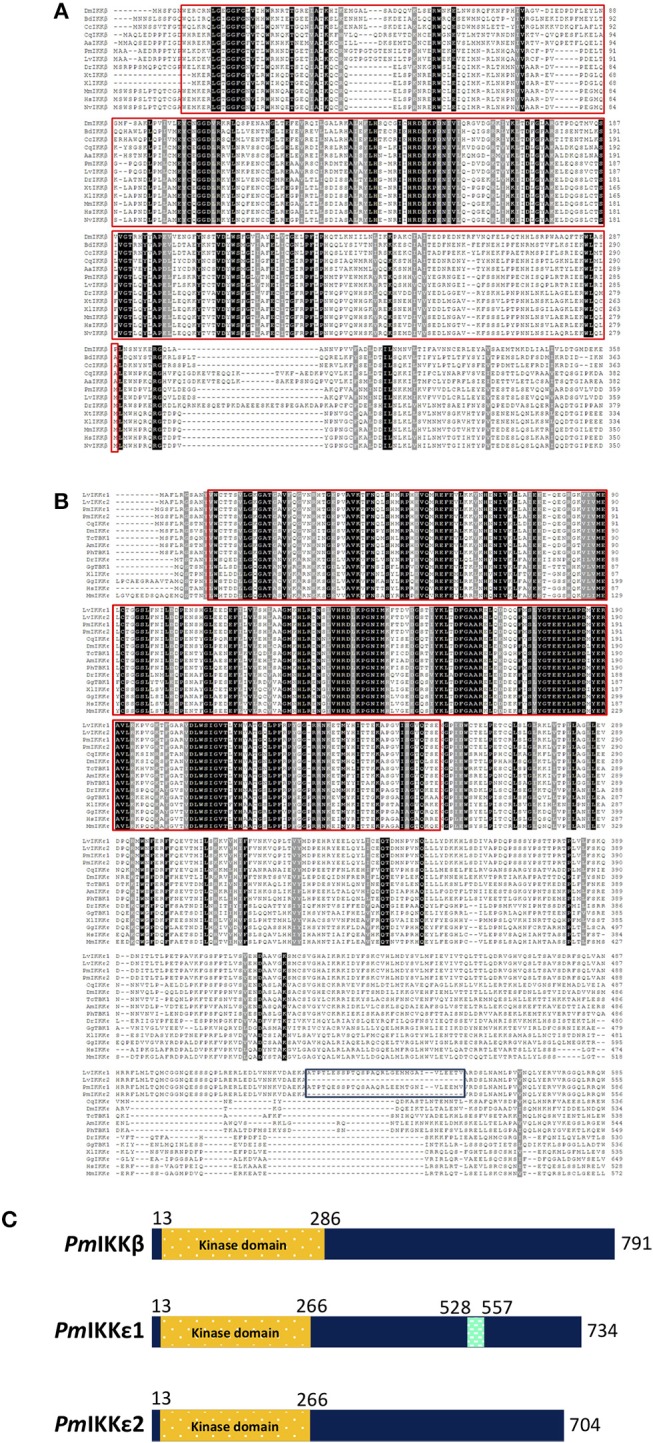
Sequence analysis of *Pm*IKK and IKK family proteins from various typical species. Multiple sequence alignments of **(A)** *Pm*IKKβ and **(B)** *Pm*IKKε with IKK-family proteins were performed using Clustal Omega (https://www.ebi.ac.uk/Tools/msa/clustalo). The amino acid sequences of *Pm*IKKβ, *Pm*IKKε1, and *Pm*IKKε2 were deduced by ExPASy-Translate tool (https://web.expasy.org/translate). Important protein motif features were predicted using Simple Modular Architecture Research Tool, SMART 8.0 (http://smart.embl-heidelberg.de). The conserved residues are shaded in black and gray. The important kinase domains at the N-termini are in the red boxes and the 30-amino acid regions of *Pm*IKKε1 and *Lv*IKKε1 are in the blue box below. **(C)** Schematic diagram of structural domain topology from PmIKKβ, *Pm*IKKε1, and *Pm*IKKε2 analyzed by SMART 8.0 program. N-terminal KDs are marked as yellow from amino acid residues 13 to 286 of *Pm*IKKβ and amino acid residues 13 to 266 of *Pm*IKKε1 and *Pm*IKKε2. The *Pm*IKKε1 contains unidentified domain from amino acid residues 528 to 557 at the C-terminus region. The amino acid sequences include; *Penaeus monodon* IKKβ (*Pm*IKKβ, MK331816); *Litopenaeus vannamei* IKKβ (*Lv*IKKβ, AEK86518); *Mus muculus* IKKβ (*Mm*IKKβ, NP_001153246); *Homo sapiens* IKKβ (*Hs*IKKβ, NP_001547); *Xenopus tropicalis* IKKβ (*Xt*IKKβ, NP_001005651); *Xenopus laevis* IKKβ (*Xl*IKKβ, NP_001085125); *Danio rerio* IKKβ (*Dr*IKKβ, NP_001116737); *Drosophila melanogaster* IKKβ (*Dm*IKKβ, AAG02485); *Culex quinquefasciatus* IKKβ (*Cq*IKKβ, XP_001865661); *Mustela putorius* IKKβ (*Mp*IKKβ, XP_004775760); *Bactrocera dorsalis* IKKβ (*Bd*IKKβ, XP_011211311); *Ceratitis capitatal* IKKβ (*Cc*IKKβ, XP_004537145); *Aedea aegypti* IKKβ (*Aa*IKKβ, XP_001656614); *Penaeus monodon* IKKε1 (*Pm*IKKε1, MK331817); *Penaeus monodon Pm*IKKε2 (*Pm*IKKε2, MK331818); *Litopenaeus vannamei* IKKε1 (*Lv*IKKε1, AEK86519); *Litopenaeus vannamei* IKKε2 (*Lv*IKKε2, AEK86520); *Culex quinquefasciatus* IKKε (*Cq*IKKε, XP_001848400); *Drosophila melanogaster* IKKε (*Dm*IKKε, NP_724278); *Apis mellifera* IKKε (*Am*IKKε, XP_396937); *Xenopus laevis* IKKε (*Xl*IKKε, NP_001089830); *Danio rerio* IKKε (*Dr*IKKε, NP_001002751); *Gallus gallus* IKKε (*Gg*IKKε, XP_428036); *Homo sapiens* IKKε (*Hs*IKKε, NP_054721); *Mus musculus* IKKε (*Mm*IKKε, EDL39711); *Gallus gallus* TBK1 (*Gg*TBK1, NP_001186487), *Tribolium castaneum* TBK1 (*Tc*TBK1, XP_969718) and *Pediculus humanus* TBK1 (*Ph*TBK1, XP_002428501).

In addition, two isoforms of *PmIKKε* including *PmIKKε1* (accession no. MK331817) and *PmIKKε2* (accession no. MK331818) were identified using RACE approach. The ORF of *PmIKK*ε*1* was 2223 bp in length encoding 740-amino acid protein with predicted molecular mass of 83.56 kDa and pI of 5.85. The *PmIKKε1* and *PmIKKε2* were 99.58% identical as the *PmIKKε2* lacked a 30-amino acid sequence at position 528 to 557 present in *PmIKKε1*, which made the predicted molecular mass and pI of 80.27 kDa and 6.03, respectively (Supplementary Figure 1B). The *Pm*IKKε1 and *Pm*IKKε2 shared 93% and 94% sequence identity with *Lv*IKKε1 and *Lv*IKKε2, respectively. Moreover, the full-length protein sequences of *Pm*IKKε1 and *Pm*IKKε2 exhibited 27% identity with *Pm*IKKβ. Protein domain characterization using SMART database revealed the N-terminal kinase domains (KDs) from amino acid residues 13 to 286 in *Pm*IKKβ and 13 to 266 in *Pm*IKKε1 and *Pm*IKKε2 (Figure 1C).

### Multiple Sequence Alignment and Phylogenetic Analysis

**Figure 2:**
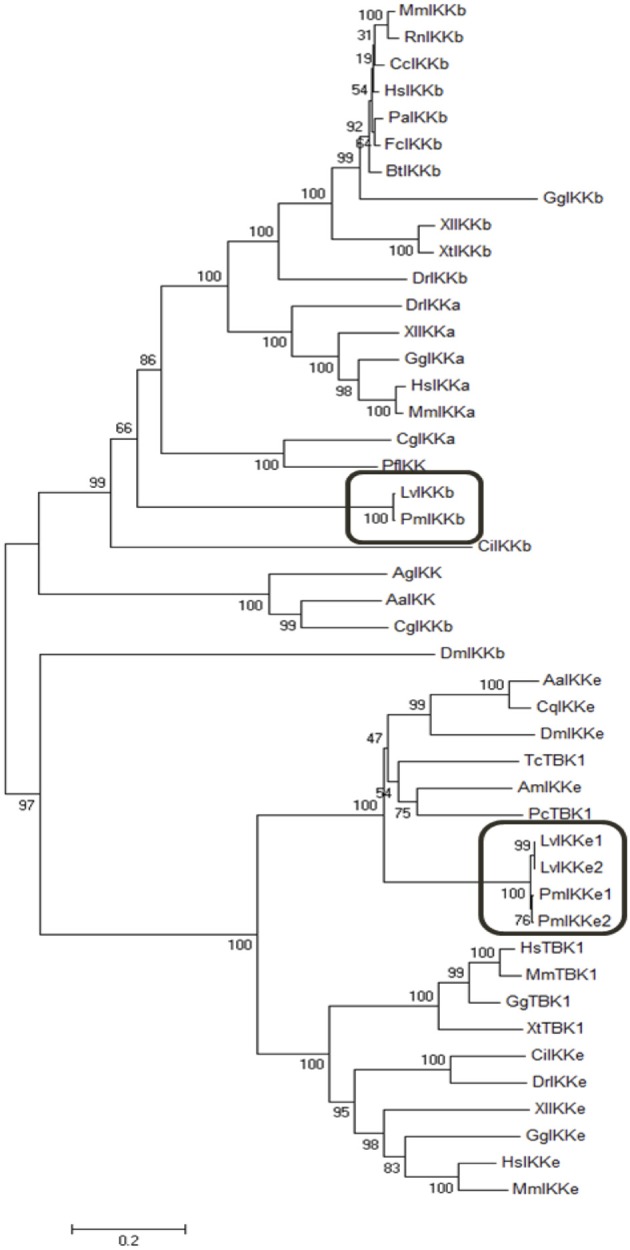
Phylogenetic analysis of IκB kinases (IKKs) and IKK-family proteins from *Penaeus monodon* and various species. The neighbor joining phylogenic tree was constructed in MEGA 7.0 software based on the amino acid sequences of IKKβ, IKKε, and IKK-family proteins from vertebrates and invertebrates. Bootstrap sampling was reiterated for 1,000 times. The deduced amino acid sequences retrieved from various species include; *Penaeus monodon* IKKβ (*Pm*IKKβ, MK331816); *Litopenaeus vannamei* IKKβ (*Lv*IKKβ, AEK86518); *Mus muculus* IKKβ (*Mm*IKKβ, NP_001153246); *Rattus norvegicus* IKKβ (*Rn*IKKβ, AAF21978); *Castor canadensis* IKKβ (*Cc*IKKβ, P_020011901); *Homo sapiens* IKKβ (*Hs*IKKβ, NP_001547); *Pongo abelii* IKKβ (*Pa*IKKβ, XP_024106853); *Felis catus* IKKβ (*Fc*IKKβ, XP_003984800); *Bos tausus* IKKβ (*Bt*IKKβ, NM_174353); *Danio rerio* IKKβ (*Dr*IKKβ, NP_001116737); *Xenopus tropicalis* IKKβ (*Xt*IKKβ, NP_001005651); *Xenopus laevis* IKKβ (*Xl*IKKβ, NP_001085125); *Cricetulus griseus* IKKβ (*Cg*IKKβ, XP_027293025); *Gallus gallus* IKKβ (*Gg*IKKβ, NP_001026568); *Drosophila melanogaster* IKKβ (*Dm*IKKβ, AAG02485); *Aedes aegypti* IKK (*Aa*IKK, EAT45468); *Anopheles gambiae* IKK (*Ag*IKK, XP_553095); *Ciona intestinalis* IKK (*Ci*IKK, XP_002125567); *Pinctada fucata* IKK (*Pf* IKK, AAX56336); *Mus* musculus IKKα (*Mm*IKKα, AAC52589); *Homo sapiens* IKKα (*Hs*IKKα, NP_001269); *Crassostrea gigas* IKKα (*Cg*IKKα, NP_001295815); *Xenopus laevis* IKKα (*Xl*IKKα, NP_001086127); *Danio rerio* IKKα (*Dr*IKKα, AAW68010); *Gallus gallus* IKKα (*Gg*IKKα, NP_001012922); *Penaeus monodon* IKKε1 (*Pm*IKKε1, MK331817); *Penaeus monodon Pm*IKKε2 (*Pm*IKKε2, MK331818); *Litopenaeus vannamei* IKKε1 (*Lv*IKKε1, AEK86519); *Litopenaeus vannamei* IKKε2 (*Lv*IKKε2, AEK86520); *Culex quinquefasciatus* IKKε (*Cq*IKKε, XP_001848400); *Drosophila melanogaster* IKKε (*Dm*IKKε, NP_724278); *Apis mellifera* IKKε (*Am*IKKε, XP_396937); *Aedes aegypti* IKKε (*Aa*IKKε, XP_001650774); *Homo sapiens* IKKε (*Hs*IKKε, NP_054721); *Mus musculus* IKKε (*Mm*IKKε, EDL39711); *Xenopus laevis* IKKε (*Xl*IKKε, NP_001089830); *Danio rerio* IKKε (*Dr*IKKε, NP_001002751); *Ciona intestinalis* IKKε (*Ci*IKKε NP_001072034); *Gallus gallus* IKKε (*Gg*IKKε, XP_428036); *Xenopus tropicalis* TBK1 (*Xt*TBK1, NP_001135652); *Homo sapiens* TBK1 (*Hs*TBK1, NP_037386); *Macaca mulatta* TBK1(*Mm*TBK1, NP_001248122); *Phalacrocorax carbo* TBK1 (*Pc*TBK1, XP_009506274.1); *Gallus gallus* TBK1 (*Gg*TBK1, NP_001186487) and *Tribolium castaneum* TBK1 (*Tc*TBK1, XP_969718).

To examine the evolutionary relationship of IκB kinases among various organisms, the deduced amino acid sequences of *Pm*IKKβ, *Pm*IKKε1, and *Pm*IKKε2 were aligned with IKK and IKK-family proteins from other species. Multiple sequence alignment performed using Clustal Omega revealed the important N-terminal kinase domains which is conserved among IKK and IKK family proteins from the examined species (Figures 1A,B). The phylogenetic analysis was performed in MEGA 7.0 software to construct an unrooted neighbor-joining phylogenetic tree based on the deduced amino acid sequences. The bootstrap sampling was reiterated for 1,000 times and demonstrated the divided clusters comprising species of mammalian, arthropod and mollusk. As the results, the *Pm*IKKβ and *Pm*IKKε from *P. monodon* were grouped with the closely related *Lv*IKKβ and *Lv*IKKε from *L. vannamei*, respectively (Figure 2).

### Tissue Expression of *PmIKKβ* and *PmIKKε*

**Figure 3:**
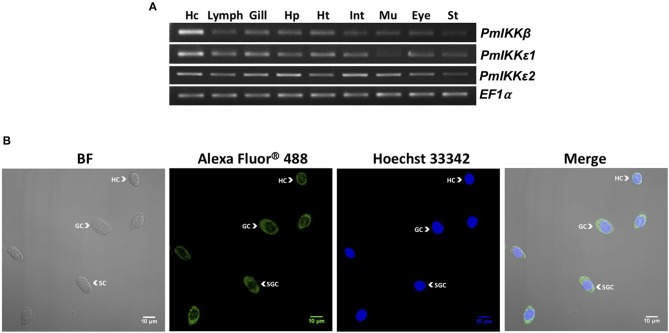
Expression of *Pm*IKKβ, *Pm*IKKε1, and *Pm*IKKε2 at the transcript and protein levels. **(A)** Tissue-specific gene expression of *PmIKK*β, *PmIKK*ε*1*, and *PmIKK*ε*2* from *Penaeus monodon*. Various tissues were collected from healthy *P. monodon* including; hemocyte (Hc), lymphoid organ (Lymp), gill (G), hepatopancreas (Hp), heart (Ht), intestine (Int), muscle (Mu), eyestalk (Eye), and stomach (St.). Total RNA was extracted for tissue distribution analysis using semi-quantitative RT-PCR. The elongation factor1-α gene (*EF-1*α) was used as an internal control. **(B)** Protein localization of *Pm*IKKε in three different types of hemocytes in bright field (BF, left), dark field (Alexa Fluor® 488 and Hoechst 33342, two at the middle) and merging (Merge, right) images including hyaline cells (HC), granular cells (GC) and semi-granular cells (SGC) from *P. monodon*. Total hemocytes were drawn from three healthy shrimp and fixed immediately in 1X PBS containing 4% paraformaldehyde. Cells were washed, counted with hemocytometer and mounted on coverslips for immunofluorescence staining. The *Pm*IKKε was detected using a monoclonal antibody specific to human IKKε. The nuclei were stained blue with Hoechst 33342 and the *Pm*IKKε was stained green with Alexa Fluor® 488. The detection was performed using an LSM700 laser scanning confocal microscope (Carl Zeiss).

Several tissues were collected from healthy *P. monodon* to investigate the mRNA expression of *PmIKKβ*, *PmIKKε1*, and *PmIKKε2*. Total RNAs were extracted from selected tissues and reverse transcribed to cDNA. The expression was observed by semi-quantitative RT-PCR using elongation factor-1α gene (*EF1-α*) as an internal control. The amplicon of 322 bp was detected from the gene-specific cDNA fragment of *PmIKKβ*. Moreover, using specifically designed primers for amplification, the amplicons of 249 bp and 230 bp from *PmIKKε1* and *PmIKKε2* were amplified distinguishably. The *PmIKKβ*, *PmIKKε1*, and *PmIKKε2* transcripts were detected in all examined tissues with high mRNA expression of *PmIKKβ* and *PmIKKε1* in hemocytes (Hc) which is an immune-related tissue. However, the *PmIKKε2* transcript was moderately expressed in hemocytes (Figure 3A).

### Protein Expression and Localization of *Pm*IKKβ and *Pm*IKKε in Shrimp Hemocytes

To examine the expression of *Pm*IKKβ and *Pm*IKKε proteins in different types of shrimp hemocytes, immunofluorescence and confocal microscopy were performed using anti-IKKβ and anti-IKKε antibodies specific to human IKKβ and IKKε, respectively. Shrimp hemocytes were collected, fixed and processed for the detection of endogenous *Pm*IKKβ and *Pm*IKKε proteins in bright field (BF), dark field (Alexa Fluor^®^ 488 and Hoechst 33342) and merging (Merge) images. Nuclei were stained blue with Hoechst 33342 while the *Pm*IKKβ and *Pm*IKKε were visualized in green with Alexa Fluor^®^ 488. The fluorescent microscopic images revealed that *Pm*IKKε protein was expressed mainly in cytoplasm and slightly in nucleus of all three types of hemocytes including hyaline cells (HC), granular cells (GC) and semi-granular cells (SGC) (Figure 3B). Unfortunately, the human anti-IKKβ antibody was not suitable for examining protein expression with fluorescent immunostaining in shrimp hemocytes as a result of weak fluorescent signal even with higher concentration used (data not shown).

### Temporal Expression of *PmIKKβ* and *PmIKKε* mRNAs After Pathogen Challenges

**Figure 4:**
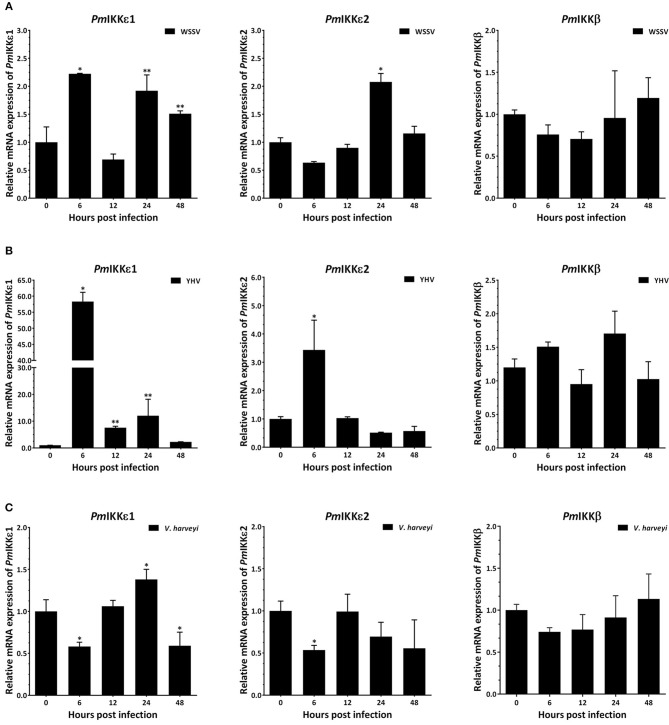
Temporal expression of *PmIKK*β, *PmIKK*ε*1*, and *Pm*I*KK*ε*2* in shrimp hemocyte upon immune challenge with WSSV, YHV and *V. harveyi*. *P. monodon* were injected intramuscularly at the third abdominal segment with 30 μl of 1X PBS as a control, 1 × 10^5^ copies of purified WSSV, 1 × 10^5^ copies of purified YHV or 30 μl of 1 × 10^6^ CFU/ml of *V. harveyi* 639. Three shrimp were randomly collected at 0, 6, 12, 24, and 48 hpi from each group and total hemocyte was obtained for qRT-PCR analysis. Expression levels of *PmIKK*β, *PmIKK*ε*1*, and *PmIKK*ε*2* in hemocytes of shrimp challenged with **(A)** WSSV, **(B)** YHV, and **(C)** *V. harveyi* was normalized with those of control PBS group and set to 1.0 at 0 hpi. Calculation of relative mRNA expression was performed according to Pfaffl method (2001) using *EF1-*α as a reference gene. Data are derived from three independently triplicate experiments and shown as the means ± SDs. Asterisks indicate significant differences of mean values (*P* < 0.05).

The transcript levels of *PmIKKβ*, *PmIKKε1*, and *PmIKKε2* were determined to investigate the effects of infection by viruses including WSSV and YHV and a bacterium *V. harveyi*. Following the infection, total shrimp hemocytes was collected for RNA isolation. Quantitative RT-PCR showed that the *PmIKKε1* and *PmIKKε2* were up-regulated at 6 and 24 h post WSSV and YHV infection compared with the PBS-injected group (Figures 4A,B). Moreover, the *PmIKKε1* was up-regulated at 24 hpi upon *V. harveyi*, whereas, down-regulation was detected at 6 and 48 hpi. The *PmIKKε2* was also slightly down-regulated by 0.5-fold at 6 hpi (*P* < 0.05) (Figure 4C). However, there was no significant difference in the expression level of *PmIKKβ* after infection with WSSV, YHV, or *V. harveyi* compared to the control PBS-injected shrimp (*P* < 0.05).

### Survival Rate of WSSV-Infected Shrimp and Viral Copy Number After *Pm*IKKβ and *Pm*IKKε Silencing

**Figure 5:**
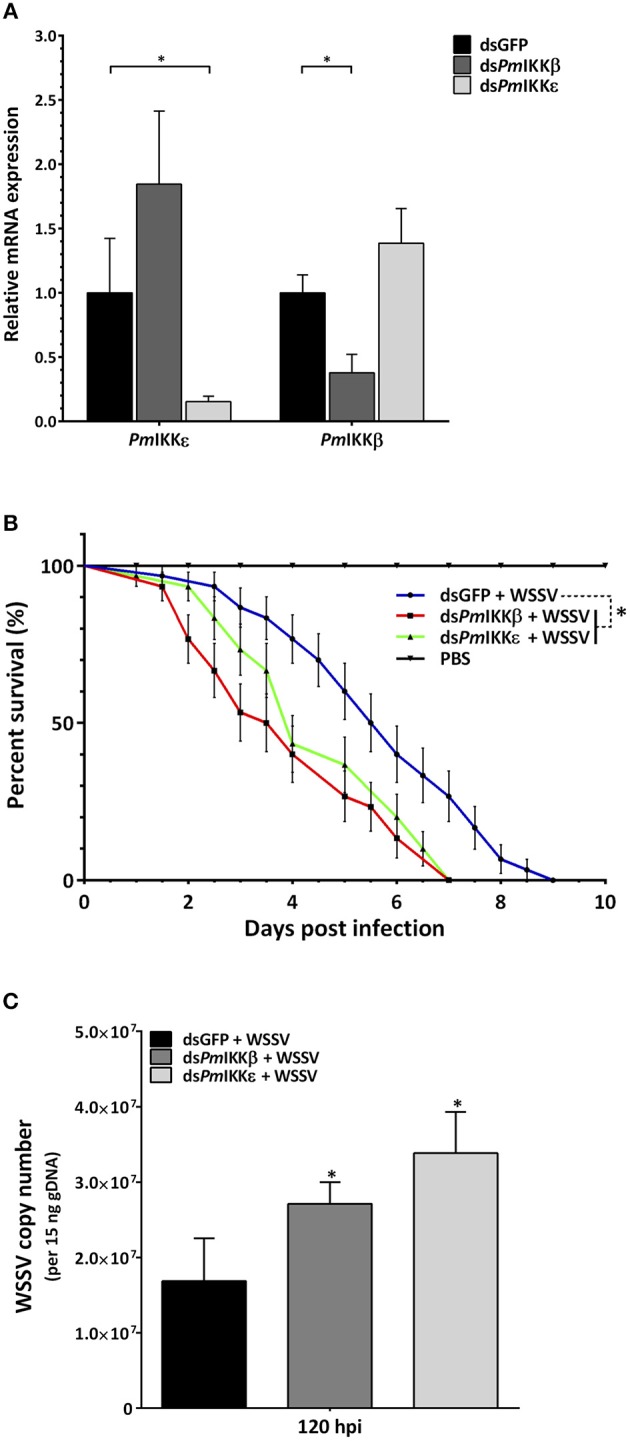
Gene silencing of *PmIKK*β and *PmIKK*ε and their effects on the survival of WSSV-challenged shrimp and the WSSV copy number. **(A)** Expression profile of *PmIKK*β and *PmIKK*ε after gene silencing by dsRNA-mediated RNAi. Shrimp were doubly injected with ds*GFP* (control), ds*PmIKK*β or ds*PmIKK*ε with an interval of 24 h followed with 1X PBS or WSSV inoculum at 6 h after the second dsRNA injection. Total hemocytes were collected at 24 h after PBS or WSSV injection for total RNA isolation and cDNA synthesis. The mRNA expression levels of *PmIKK*β and *PmIKK*ε were determined by qRT-PCR performed independently in three triplicates. The expression was normalized with *EF1-*α as a reference gene. Data are shown as the mean fold change (means ± SDs, *n* = 3) relative to a control *dsGFP*-injected group. Asterisks indicate significant differences of mean values (*P* < 0.05). **(B)** Effects of *PmIKK*β and *PmIKK*ε gene silencing on shrimp survival after challenged with WSSV and **(C)** WSSV copy number. Experimental shrimp (3–5g) were doubly injected with 10 μg/g shrimp of ds*GFP*, ds*PmIKK*β, ds*PmIKK*ε or 1X PBS with an interval of 24 h prior to WSSV or PBS injection. The mortalities were recorded daily for a period of 10 days. For WSSV copy number, the genomic DNA from gill was extracted at selected time points for qRT-PCR analysis. The experiment was performed in three independent triplicates of 10 shrimp per group. Data are shown as the means ± SDs with *n* = 3.

The dsRNA-mediated RNA interference was performed to characterize the roles of *Pm*IKKβ and *Pm*IKKε in shrimp innate immune system. Following the dsRNA injection, shrimp were randomly selected for hemocyte RNA extraction. Quantitative RT-PCR analysis was performed showing the significant suppression of *PmIKKβ* and *PmIKKε* transcripts after doubly injection of 10 μg/g shrimp dsRNA. The expression of *PmIKKβ* was significantly decreased to 0.4-fold in ds*PmIKKβ*-injected *P. monodon* compared to the level observed in the control ds*GFP*-injected group. In ds*PmIKKε*-injected *P. monodon*, the expression of *PmIKKε* was significantly decreased to 0.1-fold compared to the level observed in the control ds*GFP*-injected group (Figure 5A).

To examine the effect of *PmIKKβ* and *PmIKKε* silencing on shrimp mortality upon WSSV infection, shrimp were injected with dsRNAs as described above. Twenty-four hours following the dsRNA injection, shrimp were infected with WSSV and the cumulative mortalities were recorded daily over a period of 10 days. The results revealed that, after viral infection, *PmIKKβ*- and *PmIKKε*-silenced shrimp were susceptible to rapid death within 7 days, compared with 10 days of the control ds*GFP*-injected group (Figure 5B). In addition, both the *PmIKKβ*- and *PmIKKε*-silenced shrimp exhibited 50% cumulative mortalities at 3.5 and 4 days, respectively.

Compared to the ds*GFP*-injected group, the higher mortality rates suggest the essential roles of *Pm*IKKβ and *Pm*IKKε in shrimp immune system against WSSV infection. Moreover, the consequence of *PmIKKβ* and *PmIKKε* suppression on WSSV infection was investigated. The viral copy number of WSSV in *PmIKKβ*- and *PmIKKε*-silenced shrimp was quantified by the detection of a conserved *VP28* gene using qRT-PCR. The WSSV copy number was quantified at 120 hpi in correlation with shrimp cumulative mortalities. It was found that the viral copy number was significantly higher in the *PmIKKβ*- and *PmIKKε*-silenced shrimp, compared to the ds*GFP*-injected group (Figure 5C).

### Effect of *in vivo PmIKKβ* and *PmIKKε* Silencing on Immune-Related Genes Upon WSSV Infection

**Figure 6:**
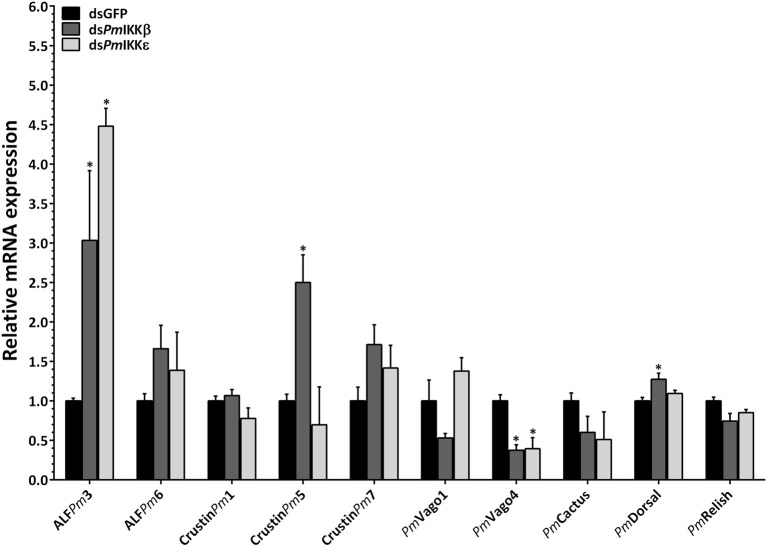
Expression of immune-related genes upon WSSV infection following *PmIKK*β and *PmIKK*ε silencing. The transcript levels of antimicrobial peptides, cytokines and transcription factors were examined upon *PmIKK*β and *PmIKK*ε silencing. Shrimp were doubly injected with ds*GFP* (control), ds*PmIKK*β or ds*PmIKK*ε followed by 1 × 10^5^ copies of purified WSSV inoculum. Total RNA was isolated from the hemocytes for qRT-PCR analysis. The expression levels of genes involved in signal transduction pathway (*PmRelish, PmCactus*, and *PmDorsal*), antimicrobial peptides (*ALFPm3, ALFPm6, CrustinPm1, CrustinPm5*, and *CrustinPm7*) and IFN-like molecules (*PmVago1* and *PmVago4*) were determined using *EF1-*α as an internal control. Data are shown as the means ± SDs from three triplicate experiments relative to the control ds*GFP* group. Asterisks indicate significant differences of mean values (*P* < 0.05).

To further investigate the functions of *Pm*IKKβ and *Pm*IKKε in shrimp immune response, the ds*PmIKKβ*- and ds*PmIKKε*-injected shrimp were subsequently either injected with 1X PBS or infected with WSSV and the mRNA levels of immune-related genes were determined by qRT-PCR. The expression of genes in signal transduction pathways (*PmDorsal, PmRelish*, and *PmCactus*), antimicrobial peptides (*ALFPm3, ALFPm6, CrustinPm1, CrustinPm5*, and *CrustinPm7*) and IFN-like genes (*PmVago1* and *PmVago4*) were analyzed. When compared with the ds*GFP* control group, the expression of *PmVago4* was significantly decreased in both *PmIKKβ*- and *PmIKKε*-silenced shrimp, while that of *PmVago1* was not affected. The expression of *PmDorsal* was increased after *PmIKKβ* suppression, whereas those of *PmCactus* and *PmRelish* remained unaffected. Moreover, the *CrustinPm5* was up-regulated in *PmIKKβ*-silenced shrimp, while the *ALFPm3* was up-regulated in both *PmIKKβ*- and *PmIKKε*-silenced shrimp. The expression of *ALFPm6, CrustinPm1* and *CrustinPm7* were not affected in both *PmIKKβ* and *PmIKKε*-silenced shrimp when compared with the ds*GFP* control group (Figure 6).

### Overexpression of Shrimp *Pm*IKKs in HEK293T and Promoter Activity Assay

**Figure 7:**
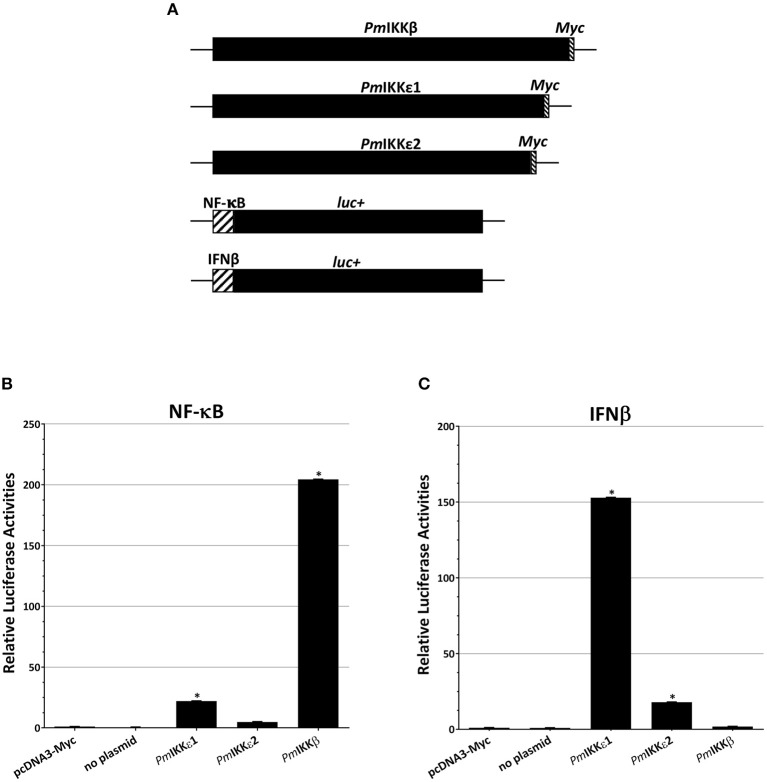
Overexpression of *Pm*IKKβ, *Pm*IKKε1, or *Pm*IKKε2 in HEK293T cells activate NF-κB or IFNβ reporters. The HEK293T cells were transiently co-transfected with luciferase reporter plasmids and control plasmid, no plasmid or a construct of protein expression plasmids as indicated **(A)**. Luciferase activities from **(B)** NF-κB and **(C)** IFNβ reporter plasmids were determined and normalized on the basis of *Renilla* expression with Dual-Glo® Luciferase Assay System (Promega). Data are shown as the means ± SDs of luciferase activities from triplicate experiments with *n* = 3. Asterisks indicate significant differences of mean values (*P* < 0.01).

To further evaluate the roles of *Pm*IKKβ and *Pm*IKKε in the regulation of shrimp cytokine-like system and NF-κB signaling, the HEK293T were transiently transfected with NF-κB or IFNβ reporter plasmids simultaneously with each construct of protein expression plasmids for *Pm*IKKβ, *Pm*IKKε1, or *Pm*IKKε2 (Figure 7A) and luciferase activities were measured. Compared with the pcDNA3-Myc control group, the overexpression of *Pm*IKKβ, *Pm*IKKε1 and *Pm*IKKε2 induced the promoter activities of NF-κB approximately 204.45-, 22.13- and 4.91-fold, respectively (Figure 7B). Moreover, only the overexpression of *Pm*IKKε1 and *Pm*IKKε2 that significantly induced the IFNβ promoter activities approximately 152.90- and 17.92-fold, respectively (Figure 7C). The results demonstrated the possible roles of *Pm*IKKε1 and *Pm*IKKε2 as the immune-stimulatory factors for an IFN-like system in shrimp. On the contrary, *Pm*IKKβ showed no significant induction on IFNβ promoter activity in HEK293T cells, compared to the control group. The result suggested that *Pm*IKKβ might serve mainly as a positive regulator of NF-κB signaling pathway in shrimp innate immune responses. In addition, the greater activation from *Pm*IKKε1 in HEK293T cells was possibly a result from the additional 30 amino acid segment whose function remained to be elucidated.

## Discussion

The IKK-NF-κB pathway has been studied and reported to regulate pro-inflammatory cytokine production, leukocyte recruitment or cell survival in vertebrates (7). It is also clear that the NF-κB is an important contributor to the immune responses and feedback control of inflammation via various mechanisms (13). The key kinase proteins, IKKs, serve as the core elements to drive the pathway upon several pathogen- or environment-induced immune responses (9, 10, 12). Although several studies in IKK-NF-κB signaling pathway have been investigated in vertebrates and fruit fly *D. melanogaster*, less attention has been paid for the mechanism in crustaceans. In this study, the IKKs from black tiger shrimp *P. monodon* (*Pm*IKKβ, *Pm*IKKε1, and *Pm*IKKε2) were identified and characterized in the innate immune system of the black tiger shrimp *P. monodon*.

From the partial nucleotide sequences from the EST database, the complete open reading frames of *PmIKKβ*, *PmIKKε1*, and *PmIKKε2* were successfully cloned and sequenced. Phylogenetic analysis demonstrated the clusters of *Pm*IKKs from *P. monodon* with *Lv*IKKs from *L. vannamei* indicating a related evolutionary relationship between the two species. The sequences of *Pm*IKKs shared high similarities approximately 95–97% at the protein level with those of *Lv*IKKs, which were reported as an invertebrate homologs of mammalian IKKα/IKKβ and strongly induced the NF-κB activity in human HEK293T cells (25). The two isoforms of *Pm*IKKε namely, *Pm*IKKε1, and *Pm*IKKε2, were highly similar to those of *Lv*IKKε from *L. vannamei*. The *Pm*IKKε1 carried an extra 30 amino acid region which was absent in the *Pm*IKKε2. Luciferase reporter assays revealed the higher IFNβ promoter activation from *Pm*IKKε1 for which the additional 30-aa region was believed to be an important element responsible for the higher NF-kB activity (25). According to the protein feature analysis from the SMART database, *Pm*IKKβ, *Pm*IKKε1, and *Pm*IKKε2 contained the important kinase domains located in their N-termini. In addition to kinase domains, the IKK and IKK-family proteins from various species also contain a NEMO-binding domain (NBD), ubiquitin-like domain (ULD) or leuzine zipper domains (LZ) which are essential for IKK complex assembly and their catalytic activities (10, 12). Therefore, the domain compositions of *Pm*IKKβ, *Pm*IKKε1, and *Pm*IKKε2 were needed to be investigated for further understanding their functions in signaling cascade.

Tissue-specific expression analysis revealed that the transcripts of *Pm*IKKβ, *Pm*IKKε1, and *Pm*IKKε2 were expressed in various shrimp tissues. The particular high mRNA expression levels of *Pm*IKKβ, *Pm*IKKε1, and *Pm*IKKε2 were found in hemocytes (Hc) which was the important tissue where several immune reactions took place (5, 26). Upon immune challenges with shrimp pathogens including viruses and a bacterium, the *Pm*IKKε1 and *Pm*IKKε2 transcripts were up-regulated whereas the *Pm*IKKβ expression was unaffected. Previous report showed that both TBK1 and IKKε were considered to be upstream of IKKβ in the NF-κB activation pathway (27). In immune mechanisms, the IKK-NF-κB signaling cascade is targeted and interrupted by various pathogens to favor the diseases (7, 25, 28). The overexpression of IKKε in human HEK293T cells was reported to induce the cRel nuclear accumulation independently of IKKβ activity (27).

Innate immune response is generally the first line of defense responding to the invading pathogens (1, 5). Recent reports suggested that the endogenous ligands from pathogens also trigger pattern recognition receptors during host infection. This activation may act to promote signal transduction mechanisms that include the production of several antimicrobial peptides (AMPs) through IKK complex (4). In terms of vertebrates, IKKε and IKK-related kinase, TANK-binding kinase 1 (TBK1) are the components which phosphorylate IRF-3 and IRF-7 triggering host antiviral responses (15, 24). Furthermore, IKKε and TBK1 may function and link to IKKα/IKKβ complexes through TANK (TRAF family member-associated NF-κB activator) with IKKγ/NEMO interactions (7). The immune challenges suggested that the *Pm*IKKs may contribute to the immune responses against dsDNA virus (WSSV), dsRNA virus (YHV) and also a bacterium *V. harveyi*.

Among several pathogens, the WSSV is one of the most destructive viruses to shrimp aquaculture worldwide leading to 100% mortality within 3–10 days after the outbreaks (29, 30). Shrimp with suppression of *Pm*IKKβ and *Pm*IKKε were more susceptible to WSSV infection as a more rapid death and higher viral copy number were detected, suggesting the essential roles in shrimp antiviral response against WSSV replication. In addition, following the *Pm*IKKβ and *Pm*IKKε silencing, the mRNA level of *PmVago4* which is an IFN-like molecule was reduced significantly. In *Drosophila melanogaster*, the dsRNA-mediated *Dm*IKK silencing inhibited the immune response leading to reduction of both IFN-β mRNA level and protein production (17). The *Dm*Vago was also reported as an antiviral molecule targeting the virion or a cytokine which subsequently triggered an infected-state in the neighboring cells (16, 17). In other arthropods, the *Cx*Vago from *Culex* was induced and secreted by West Nile virus (WNV). The *Cx*Vago acted as a homolog of interferon, activating JAK-STAT pathway and limiting virus replication in neighboring cells (22). Recently, it has been reported that shrimp Vago might function as an IFN-like molecule and shrimp might possess an antiviral mechanism similar to the IFN system (21).

In the Pacific white shrimp *L. vannamei*, the *Lv*IKKβ and *Lv*IKKε are the central regulators of the IKK-NF-κB signaling pathway and represent the points of convergence for the most signal transduction leading to NF-κB activation (25). Toll and IMD pathways are two important signaling cascades in which their components activate AMP luciferase reporters and bind to NF-κB-binding sites in the AMP promoter regions (16). Shrimp antimicrobial peptides (*Lv*Penaeidin and *Lv*Crustin) are significantly decreased upon *LvIKKβ* and *LvIKKε* silencing (25). However, in *P. monodon* the expression of *ALFPm*3, *CrustinPm5*, and *PmDorsal* were up-regulated after *PmIKK* silencing which indicated the induction of alternative immune pathways. The *ALFPm*3 transcript was decreased in *MyD88*- and *Relish*-silenced shrimp indicating that the expression of *ALFPm*3 was controlled by both Toll and IMD pathways, while that of ALF*Pm*6 was reported to be regulated only by Toll signaling pathway (31). Recently, during WSSV and *V. anguillarium* infection in Chinese shrimp *Fenneropenaeus chinensis*, a transcription factor *FcDorsal* was up-regulated (32). The transcript level of *FcPenaeidin* was decreased following the *FcDorsal* silencing suggesting that the expression of *FcPenaeidin* was regulated by Toll pathway (33).

The shrimp IMD pathway is involved for sensing of RNA viruses and Gram-negative bacteria to activate a transcription factor Relish (26). The signal-induced transcription factor Relish translocates into the nucleus and regulates the expression of shrimp penaeidins, crustins, and antilipopolysaccharide factors (ALF) (26, 34). Moreover, knockdown of the *Pm*Relish suppresses the *PmPEN5* transcript level, whereas the *PmPEN3* is slightly up-regulated. These results demonstrate that the expression of *PmPEN5* and *PmPEN3* are regulated by *Pm*Relish through shrimp IMD pathway (35). In this study, neither silencing of *PmIKKβ* nor *PmIKKε* have affected the expression of *PmRelish*. The Crustin*Pm*1 and crustin*Pm*7 are the two cationic AMPs that are identified from the hemocytes of *P. monodon*. Suppression of *PmRelish* and *PmMyD88* in regulatory pathways reveals that the expression of *CrustinPm*1 is regulated by the Toll pathway, while that of *CrustinPm*7 is regulated by both the Toll and IMD signaling pathways (36). Moreover, the *IKK*-silenced *P. monodon* showed no significant difference in the expression of *CrustinPm1, CrustinPm7*, and *ALFPm6* suggesting that the *Pm*IKKs might act as the points in several alternative regulatory factors for immune-related gene expression. In the immune response, the IKKβ compensated for the lack of IKKα to modulate the NF-κB cascade for pro-inflammatory stimulation (9, 12). These results proposed that the IKK-NF-κB pathway provided a cross-talking within proteins in the IKK family and they might not be modulated solely in one signaling pathway.

Overexpression of *Pm*IKKβ and *Pm*IKKε1 highly activated the NF-κB reporter in luciferase assay. Likewise, the IFNβ reporter was significantly induced by the *Pm*IKKε1 and *Pm*IKKε2 in HEK293T cells. The NF-κB transcription factor is a central mediator for several immune response pathways and is targeted by over a hundred microbial stimuli (7, 11). Similar to NF-κB, the IFNβ is identified as a pro-inflammatory cytokine activated in the presence of infectious diseases. The activated IFNβ stimulates the inflammatory responses in neighboring cells leading to the expression of immune-related genes involved in innate and adaptive immune responses (14, 24). The results from the reporter assays indicated the potential roles of *Pm*IKKβ and *Pm*IKKε for driving immune responses through the NF-κB signaling and cytokine-like system in shrimp defense mechanism.

More recently, IKK from *P. monodon* including *PmIKK*β and *PmIKK*ε transcripts were suppressed when a DNA sensing molecule called DEAD (Asp-Glu-Ala-Asp)-box polypeptide 41 (*PmDDX41*) was suppressed (37). The DDX41 is identified as a dsDNA-sensing receptor in mouse dendritic cells and involved in type I interferon regulation via interferon regulatory factors (IRF3 and IRF7) (38). The silencing of *PmDDX41* affects several downstream immune genes including transcription factors and AMPs (37). These results demonstrate the signaling cascade generated from the *Pm*DDX41 as a sensing molecule through *Pm*IKKβ and *Pm*IKKε for shrimp immune response. In vertebrates, signal transduction induced by stimuli leads to IKK complex formation and domain organization of catalytic subunit which are critical for its activity. The induced IKK complex serves as a signaling hub for the NF-κB signaling cascade which associates with other physiological processes (9, 10). However, the in-depth mechanism responsible for the activation of NF-κB signaling pathway in shrimp by *Pm*IKKβ and *Pm*IKKε is still unclear. Therefore, it is interesting to further investigate the mechanism of how the shrimp IKK function in innate immune system or initiate the production of antiviral molecules.

In summary, the *Pm*IKKβ, *Pm*IKKε1, and *Pm*IKKε2 were identified and characterized in shrimp antiviral responses. The *PmIKK*ε*1* and *PmIKK*ε*2* but not *PmIKK*β were up-regulated in responses to viruses and a bacterium suggesting the essential roles against pathogen infection. Suppression of *PmIKK*β and *PmIKK*ε resulted in subsequent reduction of an IFN-like *PmVago4*. Vago appears to function as a cytokine that acts in a similar manner to mammalian interferons (22). Upon, virus infection in fruit fly *Drosophila*, a specific transcriptional response including the induction of Vago was initiated (38). In *Aedes aegypti* mosquito, the *Ae*Vago1 was found to be induced by dengue virus and may contribute to limit viral replication (39). However, there was no significant induction for *PmVago1* in the black tiger shrimp. Meanwhile in *Drosophila* S2 cells, *Lv*Vago4 from *L. vannamei* was significantly induced by *Lv*IRF but no induction of *Lv*Vago1 was observed (21). One possible explanation is that, *PmVago1* may not directly play a role in the IKK-NF-κB-mediated antiviral response of shrimp and is an issue for further investigation. Moreover, the *PmIKKβ*- and *PmIKKε*-silenced shrimp were more susceptible to WSSV infection indicating that the *Pm*IKKβ and *Pm*IKKε participated in the regulation of viral infection. In addition, the overexpression of *Pm*IKKβ and *Pm*IKKε in HEK293T cells enhanced the NF-κB and IFNβ promoter activities, respectively. Therefore, this study demonstrated the potential roles of *Pm*IKKs in an IFN-like system through *PmVago4* and cross-talking between signaling transductions for regulating antiviral responses in shrimp.

## Data Availability

The raw data supporting the conclusions of this manuscript will be made available by the authors, without undue reservation, to any qualified researcher.

## Ethics Statement

This study was carried out in accordance with the recommendations of animal use protocol, approved by Chulalongkorn University Animal Care And Use Committee (CU-ACUC).

## Author Contributions

AT and PA contributed to the study, experiment designs, and funding acquisition. TK participated in the review of the study, collaboration, and provided a part of the resources. ZN performed *in vivo* and *in vitro* experiments and analyzed the data. AT reviewed the results, wrote, edited and approved the final version of the manuscript.

### Conflict of Interest Statement

The authors declare that the research was conducted in the absence of any commercial or financial relationships that could be construed as a potential conflict of interest.
