# Supplementary material for: Penaeus monodon IKKs Participate in Regulation of Cytokine-Like System and Antiviral Responses of Innate Immune System
Source: Front Immunol. 2019 Jun 25;10:1430. doi: 10.3389/fimmu.2019.01430 (PMC6604761; doi:10.3389/fimmu.2019.01430)
Supplement: Supplementary file 1 [file Data_Sheet_1.PDF]

Supplementary Material

Penaeus monodon IKKs participate in regulation of cytokine-like system and antiviral responses of innate immune system

Zittipong Nhnhkorn, Piti Amparyup, Taro Kawai, Anchalee Tassanakajon*
***Correspondence:** Professor Dr. Anchalee Tassanakajon : anchalee.k@chula.ac.th

A ***Pm*IKKβ**

```
 M  A  A  A  E  D  R  P  P  T  Y  P  W  L  K  D  K  V  L  G  T  G  G  F  G  T  V  T  L  W     30
atggcagcagcagaagaccgcccaccgacataccccctggcttaaagacaaggttttggggactggtggatttggtacagtaacattatgg     90

 R  H  N  D  T  G  E  T  I  A  L  K  K  C  R  W  G  T  P  G  T  G  T  E  N  I  L  T  P  K     60
cgtcacaacgatactggagaaactattgccctgaaaaaatgccgctggggaacaccaggaactggcacagaaaatatcctaaccccaaag     180

 H  V  E  R  W  E  K  E  V  E  I  M  N  R  L  N  H  Q  A  V  V  R  C  F  P  V  P  D  E  L     90
catgttgagcgttgggaaaaggaagtagagatcatgaatcgcttaaatcatcaagcagttgtgagatgtttcccagttcccgatgagcta     270

 T  G  P  Q  G  D  L  P  M  L  C  M  E  Y  C  S  G  G  D  L  R  K  V  L  N  K  P  E  N  C     120
actgggcctcagggagatcttcccatgctttgtatggaatactgtagtggtggtgaccttcgcaaggttttaaataagccagagaattgc     360

 C  G  L  R  E  A  A  V  R  S  C  I  R  D  M  T  E  A  V  A  Y  L  H  S  M  R  I  I  H  R     150
tgtggtttacgggaagctgctgtccgttcctgcataagagacatgactgaagctgtggcctaccttcactctatgcgtatcattcatcga     450

 D  L  K  P  E  N  I  V  L  Q  D  V  D  G  K  T  V  Y  K  L  I  D  L  G  Y  A  K  E  L  E     180
gatctgaagcctgagaacattgttctacaggatgttgatgggaaaacagtctacaaacttattgaccttggatatgcgaaagaattagaa     540

 Q  S  S  V  C  T  S  F  V  G  T  L  Q  Y  L  A  P  E  L  F  L  S  K  R  Y  T  C  T  V  D     210
caaagcagtgtctgcacctcctttgttggcacgctccagtacttggctccagaactgttcctcagcaaacgatacacttgcactgttgac     630

 Y  W  S  L  G  L  V  T  H  E  I  I  T  G  I  R  P  F  L  P  N  M  T  P  V  E  W  M  K  R     240
tactggagtctaggtcttgttacacatgaaattattacaggaattcgcccctttcttccaaatatgactccagttgaatggatgaagcgt     720

 V  R  T  K  Q  S  H  H  V  C  V  Y  E  G  R  N  G  E  I  Q  F  S  S  H  M  F  P  E  C  H     270
gtacgcacaaagcagtcccatcatgtatgtgtatatgaaggtcgcaatggagaaattcagttcagttcccacatgtttccagaatgtcat     810

 I  S  Q  P  L  R  T  R  I  E  E  W  L  R  I  M  L  E  W  D  P  V  L  R  G  Q  V  L  D  E     300
atctcacagcctctgagaacaagaatagaggagtggttgcgcatcatgctggagtgggaccctgttttgcgaggccaggtgctggacgag     900

 G  G  A  K  Q  F  V  A  F  N  M  I  N  D  I  L  N  K  K  M  I  K  V  F  V  V  D  L  C  R     330
ggcggggccaagcagtttgtggctttcaacatgatcaacgacattttaaacaagaagatgattaaagtgttcgtagtggacttgtgtcgc     990

 L  L  E  Y  E  V  T  E  S  T  S  L  S  E  V  Q  Q  W  V  A  R  D  S  G  V  L  V  D  D  Q     360
ttgctggaatatgaagtgacagagtccacatccctgtctgaggtgcagcagtgggttgcacgtgacagtggggtgttggtggatgaccaa     1080

 R  P  L  L  P  R  G  Q  P  P  D  P  T  R  P  A  I  Q  C  W  A  P  P  D  E  D  E  W  L  L     390
aggcccctactcccacgtgggcagcccccgaccccactaggcctgccatccagtgctgggcccctccggatgaagatgaatggttgttg     1170

 Y  I  F  A  E  G  M  T  R  P  Q  V  P  P  H  F  P  P  L  V  E  A  M  L  R  E  P  R  T  A     420
tatatatttgctgagggcatgacgcgaccacaggtgccaccacactttccacctttagtagaggcaatgttaagagaaccccgaacggct     1260

 V  E  Y  Q  T  Q  R  R  M  W  A  H  A  V  F  F  L  H  R  E  A  R  L  L  T  L  L  T  Q  A     450
gtcgaataccagacgcagcgtagaatgtgggcacatgctgtgttcttccttcatcgggaagcccgtctccttaccctgttaacgcaagca     1350

 Q  K  V  S  M  L  H  L  M  S  G  H  A  Q  L  T  K  T  G  Q  R  M  L  S  D  I  A  K  L  Q     480
cagaaagtttcaatgcttcacctaatgtctggccatgctcagttaaccaaaaccggccagagaatgcttagtgacattgcaaaacttcag     1440

 A  R  H  H  L  F  M  E  A  L  N  T  D  L  D  Y  Y  D  E  Q  A  S  S  G  R  L  T  S  E  K     510
gcccgtcaccatctcttcatggaggcgctcaacacggacctcgactactatgatgagcaggcttcttcaggacgccttacctcagagaag     1530

 L  Y  S  G  W  R  E  M  G  E  V  T  L  R  Q  V  H  A  V  V  E  R  V  Q  Q  L  E  G  S  L     540
ctatacagtggctggagagaaatgggagaggtaaccctaagacaagtacatgctgtggtcgaacgtgtccagcaactagaaggctcactc     1620

 T  A  L  N  T  R  I  L  E  L  Q  C  S  P  F  A  R  A  R  A  I  D  S  L  D  S  V  L  T  A     570
actgccttaaacactcgcatcctggaactacagtgttcaccatttgcccgagcaagagctattgattctctggattctgtattaacagct     1710

 G  E  D  H  Y  C  N  L  R  R  R  N  K  E  Q  R  A  T  P  H  D  N  T  D  M  C  K  L  L  L     600
ggagaagaccattattgtaatctaagaagaaggaataaagagcagcgtgctacaccacatgataatacagacatgtgcaagttgctgcta     1800

 Q  A  L  R  K  R  D  R  L  Q  Q  D  L  Y  K  H  V  E  K  Q  S  E  C  C  S  E  V  A  A  L     630
caagctttgagaaaaagggacagattgcaacaagatctttataaacacgtagagaaacagagtgaatgttgcagtgaagtagctgctctc     1890

 S  S  P  L  E  A  V  L  Q  D  A  A  R  T  A  Q  H  I  S  S  L  Q  K  Q  R  Q  K  D  I  W     660
tcttcaccattagaagcagttttgcaagatgcagcaagaaccgcacaacacatatcttcacttcagaagcaacgccagaaggacatttgg     1980

 K  I  M  E  I  A  I  N  H  S  R  T  A  G  T  A  A  G  M  A  Q  A  P  Q  T  I  P  N  A  S     690
aagatcatggaaattgcaattaaccacagccgtactgcggggacagcagctggaatggcccaggcgccccagaccattccaaatgcttcg     2070

 Q  L  P  K  K  P  P  S  P  A  V  L  S  S  L  N  N  L  L  E  K  S  K  K  E  S  D  A  I  I     720
cagttgccaaagaaacccccatcaccagcagtcttgagctcactcaataatttgcttgaaaagtcaaagaaggaatcagatgccattata     2160

 A  E  N  R  A  L  R  C  Q  M  V  E  M  L  S  G  N  V  N  S  N  L  V  I  G  A  A  R  S  P     750
gctgaaaatcgtgctcttcgttgccagatggttgagatgctctcaggaaatgtaaactccaaccttgtaataggcgcagccaggtctcca     2250

 T  G  L  S  P  P  V  T  A  D  G  L  M  L  P  P  A  L  P  E  K  R  T  P  S  P  T  S  Q  A     780
actggcctgagccctccagtcacagcagatggcctcatgctgccaccagccttgcctgagaagaggactccttccccaaccagtcaagca     2340

 A  L  N  K  K  A  V  E  T  S  L  *                                                            791
gcactgaataagaaggcagttgaaacttccttgtag                                                          2376
```

B ***PmIKKε***

```
M G S F L R G S A N Y V W C T T S V L G K G A T G A V F Q G   30
atgggttcatttctgcgaggatcagccaactatgtctggtgtacgacttctgtcttggggaaggggggccacgggagctgtctttcagggc   90

V N R H T G E P V A V K T F N Q L S H M R P H E V Q M R E F   60
gtcaacaggcatacgggagaaccagttgctgtcaagacgtttaatcagctctcacacatgcggccacacgaagtacaaatgcgtgagttt   180

E V L K K V N H E N I V K L L A I E E E Q E G R G K V I V M   90
gaagtacttaagaaggtcaaccatgagaatattgtaaaactcctggctatagaagaagagcaagaaggccggggggaaggtgattgtgatg   270

E L C T G G S L F N I L D D P E N S H G L E E D E F I L V L   120
gagctttgtaccggaggctcactcttcaacattctggacgatccagaaaatagtcatggcctggaggaggatgagtttatcttggttctg   360

S H L A A G M K H L R D N S L V H R D L K P G N I M K F T D   150
tcacatcttgctgcagggatgaaacatttgagggacaatagtctagtacatcgtgatctcaaaccggggaacatcatgaagtttacagat   450

V D G S T I Y K L T D F G A A R E L Q D D Q Q F M S L Y G T   180
gtcgacggatctactatatataagttaacagatttggtgctgctcgagaattgcaagatgaccagcagttcatgtctctatatggaaca   540

E E Y L H P D M Y E R A V L R K P V G K T F G A R V D L W S   210
gaagagtatttgcaccccgacatgtatgaacgtgcagtgctcagaaaacctgtcgggaagacctttggagcccgggtggatctgtggtca   630

I G V T L Y H V A T G Q L P F R P Y G G R R N K E T M Y H I   240
ataggtgtgacactttaccacgtggccacaggtcagcttcctttccggccgtatggaggtcggcgaaacaaagagaccatgtaccatata   720

T T E K A P G V I S G V Q T S E N G P I D W C T E L P E T C   270
acaacggagaaggccccaggagtcatatcaggtgtacagacttcagaaaacgggccaattgactggtgcacggagctgcctgaaacttgc   810

R L S L G L R K L V T P L L A G L L E V D P Q R M W N F E R   300
cggttgagcctggggctccgtaagttggtaactcctctactagcaggccttcttgaagttgatccccagagaatgtggaactttgaaagg   900

F F Q E V T M I L S K K V V H I F F V N K V Q P I T V Y M D   330
ttcttccaggaagttactatgatactgagcaagaaagtggttcacatcttcttcgtaaacaaggtgcagcctattacggtatacatggat   990

P E H R Y E E L Q Y L I C E Q T D M N P V N Q L L L Y D K K   360
ccggaacataggtatgaagaactgcaatacctgatttgtgaacagacagacatgaatccagtcaaccagcttctgctctatgacaagaaa   1080

H L S D I V A P D Q P S S S Y P S T T P R T P L V L F S K Q   390
cacttgagtgacattgtggctccagaccagccgtcttcttcgtatccgtcaacaactcctcgaacgccgttggttctcttctcaaaacaa   1170

D D D I T L T L P E T P A V K F G S F P T L V S V E H D A A   420
gatgatgacatcacactcactctaccagaaaccccggctgttaaatttggaagcttcccaactttggtaagtgtagaacatgatgctgca   1260

V G K S M C S V G H A I K R K I D Y F S K C V H L M D Y S V   450
gtgggaaaagtcaatgtgttcagttggccatgctattaagcgcaagatcgactacttctcaaaatgtgtccacctgatggattatagtgtt   1350

L M F I E V I V T Q L T T L Q D R V G H V Q S L T S A V S D   480
ctcatgttcatcgaagtgattgtcacccaattaacgactctgcaagaccgtgttggccacgtccagtcccttacatcagctgtcagtgat   1440

R F S Q L V A N H R R F L M L T Q M C G G N Q E S S S Q P L   510
cgttttagtcagttggtagccaatcacagaagattccttatgttaactcagatgtgtggaggaaaccaggagagcagctctcaacccta   1530

R E R L E D L V N N K V D A E K A A T P T Q E S S P T Q S A   540
agagaacgtctagaggatctggtcaacaacaaagttgatgctgagaaagctgccacaccaacccaggagtcatcacccacccaatcagca   1620

A Q R L E E M T G N I V L E E M V V R D S L N A M L P V V N   570
gcccagaggcttgaagagatgacgggaaacatagtcctcgaggagatggtggttcgcgactccctcaatgccatgctgccagttgtgaac   1710

Q L Y E R V V R G G Q L R R Q W Q Q A G N N A V A V E R A P   600
cagctgtacgagagggtggtccgaggaggccagctgcgtcgtcagtggcagcaggctggaaacaatgccgtagctgtggagagagcgcca   1800

N K A S T Y V T K L R E S W Q H L L R D R A A R T L T F N D   630
aataaagcctctacttacgtcaccaaactcagggagtcttggcagcacttgctcagagatagagcagcaagaacactaacatttaacgat   1890

E Q F H L L E K M K M K E T A K S L E T L L A S V T A T L H   660
gagcagttccacttgctcgagaagatgaaaatgaaagagacggcgaagtccttagaaaccctcctggcctccgtcacagctacacttcac   1980

H T T D N L A D W C K V A K V Q R V Q T E I E E A D V E K H   690
cacactacagataacttggccgactggtgcaaagtcgccaaagtccagcgagtccagacggagattgaagaggcggacgttgagaagcac   2070

E G L L S S F Q D T L G N T E D Q Y H Q T L S G L L A A I K   720
gaggggctgttgtcttctttccaggacaccttaggcaatacagaggaccagtaccaccaaaccctctctggactcctggcagccattaag   2160

D K K L Q D D P R L Q T E N P A A A L E *   740
gacaagaagttgcaggacgacccgagattgcaaactgagaatccagcggccgcactcgagtga   2223
```

Supplementary Figure 1 Nucleotide and deduced amino acid sequences of *PmIKKβ*, *PmIKKε1* and *PmIKKε2* genes from *Penaeus monodon*. The ORFs of (**A**) *PmIKKβ* and (**B**) *PmIKKε1* and *PmIKKε2* were cloned and sequenced successfully. The start codons (atg) are bold and stop codons (tag, tga) are indicated with asterisks (*). The 90-bp nucleotide sequence which is absent in *PmIKKε2* is boxed. The important kinase domains (KDs) were predicted using SMART program. The N-terminal KDs are underlined from amino acid residues 13 to 286 in *Pm*IKKβ and 13 to 266 in *Pm*IKKε1 and *Pm*IKKε2.
